# Atlas of Venereal and Skin Diseases

**Published:** 1889-06

**Authors:** 


**Atlas of Venereal and Skin Diseases,** comprising original illustrations and selections from the plates of Prof. Kaposi, of Vienna; Dr. J. Hutchinson, of London; Prof. I. Neumann, of Vienna; Profs. A. Fournier and A. Hardy, and Drs. Ricord, Cullerrier, Besnier and Vidal, of Paris; Prof. Leloir, of Lille; Dr. P. A. Morrow, of New York; Dr. E. L. Keyes, of New York; Dr. A. R. Robinson, of New York; Dr. J. Nevius Hyde, of Chicago; Dr. Henry G. Piffard, of New York; Dr. F. N. Otis, of New York, and others; with original text by PRINCE A. MORROW, A. M., M. D., Clinical Professor of Venereal Diseases, formerly Clinical Lecturer on Dermatology in the University of the City of New York; Surgeon to Charity Hospital. Fasciculus X., XI., XII., XIII. New York: William Wood & Co. 1889.

This excellent work of Venereal and Skin Diseases has been favorably noticed in previous numbers of the JOURNAL, and the

Fasciculi, as they appear, fully warrant the high encomiums which the medical press have bestowed. If clinical instruction cannot be attained in dermatology, a department of medicine always obscure to the practitioner and also to the student, we do not know another work which furnishes so clear a description in its text and so accurate a delineation in its plates. Indeed, the plates are taken from actual cases, are naturally colored, clear and distinct, and give a method of study which promises better results than any work with which we are acquainted. The illustrations are taken from the best authors, and are reliable and trustworthy. We cannot give a review of such a work. The subjects portrayed in the plates will afford a clearer idea of the scope of the work.

The Tenth Fasciculus contains five excellent plates on Eczema of the Palm, Psoriasis of Palm, Eczema Rubrum, Eczema Seborrhoicum—dry, scaly and moist forms—Impetigo Figurata and Contagiosa, Dermatitis, Exfoliativa, Pityriasis Rubra, Dermatitis Medicamentosa, Eruptions from Iodide and Bromide Potassium

The Eleventh Fasciculus contains five plates on Herpes Zoster, Febrilis and Progenitalis, Herpes Zoster, Dermatitis Herpetiformis, Pemphigus Vulgaris and Foliaceus, Purpura Simplex and Thrombotica.

The Twelfth Fasciculus contains five plates on Psoriasis of Body, Hand and Arm, Lichen Planus, Ruber, and Ruber Moniliformis, Acne Vulgaris and Rosacea, Moluscum Epitheliale, Verruca Senilis.

The Thirteenth Fasciculus contains five plates illustrating Elephantia-is of Leg and Scrotum, Leucoderma, Alopecia Areata, Keloid, Fibroma, Xanthelasma, Rhinoscleroma, Xeroderma Pigmentosum.

The text, which is from the pen of Dr. Morrow, furnishes a clear description of symptoms and treatment of the diseases illustrated by the plates. Altogether, the work, thus far, fulfils the purpose announced in its inception by the publishers, and is alike creditable to their enterprise and the ability of the accomplished author.

